# RETRACTION: Effects of Accelerated Rehabilitation Surgical Care on the Surgical Site Wound Infection and Postoperative Complications in Patients of Lung Cancer: A Meta‐Analysis

**DOI:** 10.1111/iwj.70166

**Published:** 2024-12-15

## Abstract

**RETRACTION:** FuL.‐N. , and XiaoB. , "Effects of Accelerated Rehabilitation Surgical Care on the Surgical Site Wound Infection and Postoperative Complications in Patients of Lung Cancer: A Meta‐Analysis,” International Wound Journal 21, no. 4 (2024): e14551, 10.1111/iwj.14551.38084011 PMC10961038

The above article, published online on 11 December 2023, in Wiley Online Library (http://onlinelibrary.wiley.com/), has been retracted by agreement between the journal Editor in Chief, Professor Keith Harding; and John Wiley & Sons, Ltd. Following an investigation by the publisher, all parties have concluded that this article was accepted solely on the basis of a compromised peer review process. The editors have therefore decided to retract the article. The authors did not respond to our notice regarding the retraction.

**RETRACTION:** L.‐N. Fu , and B. Xiao , "Effects of Accelerated Rehabilitation Surgical Care on the Surgical Site Wound Infection and Postoperative Complications in Patients of Lung Cancer: A Meta‐Analysis,” International Wound Journal 21, no. 4 (2024): e14551, 10.1111/iwj.14551.38084011 PMC10961038

The above article, published online on 11 December 2023, in Wiley Online Library (http://onlinelibrary.wiley.com/), has been retracted by agreement between the journal Editor in Chief, Professor Keith Harding; and John Wiley & Sons, Ltd. Following an investigation by the publisher, all parties have concluded that this article was accepted solely on the basis of a compromised peer review process. The editors have therefore decided to retract the article. The authors did not respond to our notice regarding the retraction.

